# Obesity Promotes EAE Through IL-6 and CCL-2-Mediated T Cells Infiltration

**DOI:** 10.3389/fimmu.2019.01881

**Published:** 2019-08-27

**Authors:** Zhe Ji, Shuai Wu, Yaru Xu, Jingjing Qi, Xiaohui Su, Lei Shen

**Affiliations:** ^1^Translational Medicine Research Center, Ruijin Hospital North, Shanghai Jiao Tong University School of Medicine, Shanghai, China; ^2^Shanghai Institute of Immunology, Department of Immunology and Microbiology, Shanghai Key Laboratory for Tumor Microenvironment and Inflammation, Shanghai Jiao Tong University School of Medicine, Shanghai, China

**Keywords:** obesity, EAE, infiltration of T lymphocyte, CCL-2, IL-6

## Abstract

Growing evidence suggests that obesity is associated with the susceptibility and disease severity of multiple sclerosis. The chronic inflammation induced by obesity is believed to contribute to this process. However, the immune mechanisms connecting obesity to the prevalence and pathogenesis of MS are poorly defined. In this study, we show that high fat diet (HFD)-induced obese mice developed an exacerbated EAE as indicated by higher clinical scores and more severe pathological changes in spinal cord than the control mice fed with normal diet (ND), following immunization with myelin oligodendrocyte glycoprotein (MOG) 35–55 peptide. The exacerbation of EAE in HFD mice was associated with enhanced microglial activation and increased expansion of Th1 and Th17 cells. The HFD mice also showed aggravated disease in an adoptive T cell transfer EAE model. Mechanistically, HFD augmented the expression level of IL-6 and CCL-2 both in serum and brain, and blockade of IL-6 and CCL-2 signal ameliorated EAE with reduced T cells infiltration in CNS. Taken together, our results suggest that obesity promotes CNS inflammation in EAE through IL-6 and CCL-2 mediated the inflammatory cells infiltration.

## Introduction

The incidence of obesity has increased dramatically worldwide, with an estimated 600 million adults and 100 million children are obese (1), and it has been considered a major public health problem (2). Epidemiological evidence has linked obesity to a variety of metabolic diseases, such as type 2 diabetes, cardiovascular disease, and cancer (3, 4). Recent studies indicate that obesity even predisposes individual multiple sclerosis (MS). There are several studies in the last decade demonstrating that early childhood and adolescent obesity are significant risk factors for MS susceptibility (5–8). Therefore, the rise in obesity prevalence could contribute to the higher rates of MS patients.

Multiple sclerosis is a chronic inflammatory demyelinating disease of the central nervous system (CNS) that affects about 2.5 million individuals worldwide and lacks of curative treatments (9). Experimental Autoimmune Encephalomyelitis (EAE) is the most widely used animal models to define the immunopathogenic mechanisms involved in the development of MS (10). The inflammation is one of the hallmarks of the pathogenesis in both MS and EAE, which is characterized by auto-reactive T cells and macrophage infiltration in CNS. The disease is initiated when antigen-specific Th1 and Th17 cells enter CNS. Upon encountering antigens, these T cells are re-stimulated, and release cytokines to attract inflammatory leukocytes accumulation, leading to the explosive inflammatory cascade, and tissue destruction including demyelination and axonal injury (11, 12). EAE can be induced either by immunizing animals with myelin antigens (actively induced EAE) or by transferring encephalitogenic T cells (adoptive transfer EAE) (13–15). MS and EAE develop in individuals that are genetically susceptible and require additional exposure to environmental factors (16, 17).

Obesity is associated with the development of chronic low-grade inflammation, characterized by M1 macrophage accumulation and elevated adipokines and cytokines, including leptin, TNF-α, IL-6, IL-1β, and CCL-2 (18). In addition to macrophages, our previous work had shown that adaptive immune cells including T cells and B cells, also infiltrate visceral adipose tissue (VAT), resulting in the chronic release of pro-inflammatory cytokines IFN-γ and IL-17. These adaptive immune cells can further promote VAT macrophage M1 polarization and subsequent TNF-α release, therefore worsen adipose tissue inflammation (19, 20). The obesity-induced inflammation is not only the major driver for the development of insulin resistance and Type 2 Diabetes, but also a potent accelerator for many inflammatory diseases (21, 22). The pre-existing inflammatory state in obesity may have immune-enhancing effect to worsen local inflammation in those autoimmune diseases. In this study, we demonstrated that HFD-induced obesity promoted the EAE severity by enhancing the CNS inflammation featured by pathogenic T cells activation in an CCL-2 and IL-6 dependent manner. Our finding reveals a critical role of CCL-2 and IL-6 in obesity-associated CNS inflammation and that may be potential therapeutic targets for prevention and treatment of obesity-related MS.

## Materials and Methods

### Peptides and Antibodies

Rat MOG35–55 peptides were purchased from Biosynth International (Naperville, IL, USA) and the purity of the peptide was >95%. The sequence of MOG35–55 was MEVGWYRSPFSRVVHLYRNGK. FITC anti-mouse CD3, PE/Cy7 anti-mouse CD4, APC/Cy7 anti-mouse CD44, PerCP/Cy5.5 anti-mouse CD11b antibodies, and LEAF Purified anti-mouse IL-6 were purchased from BioLegend (San Diego, CA,USA). Alexa Fluor700 anti-mouse CD45.2, PE anti-mouse CD69, APC anti-mouse CD25, PE anti-mouse IL-17A, and APC anti-mouse IFN-γ antibodies were purchased from eBioscience (San Diego, CA, USA). PE/Cy5 anti-mouse CD8α antibodies were purchased from BD Pharmingen (Basel, Switzerland). Goat anti-mouse ionized calcium-binding adaptor molecule-1 (IBA1) antibody was obtained from abcam (Cambridge, UK). Rat anti-mouse CD45, Rabbit anti-mouse CCL-2 antibody, and CCL-2 (rat recombinant) were purchased from AbD Serotec (Raleigh, NC, USA). Rabbit anti-mouse CCR2 antibody was purchased from Abcam (Cambridge, UK). 2-((1-benzyl-indazol-3-yl) methoxy)-2-methyl propionic acid (Bindarit) was synthesized by and obtained from Angelini (Angelini Research Center-ACRAF, Italy).

### Animal Models

#### Animals and Diets

C57BL/6J mice were obtained from Shanghai SLAC Laboratory Animal Co. Ltd (Shanghai, China) at 5–6 weeks of age and housed in ventilated Plexiglas cages within a pathogen-free barrier facility that maintained a 12-h light/dark cycle. The procedure for animal surgery was performed in accordance with the Guidelines of Animal Care and Use Committee of Shanghai Institute of Immunology, Shanghai Jiao Tong University School of Medicine. Mice had free access to autoclaved water and irradiated pellet food. High fat diet (HFD) that contains approximately 60% of calories from fat was purchased from Research Diets Inc. (D12492, New Brunswick, New Jersey, USA). All other mice were fed a standard pellet diet that derived about 5% of calories from lipids (SLAC Lab Diet; Shanghai, China).

#### Active Immunization Model of EAE

Induction of EAE was performed as previously described (23). Briefly, mice were subcutaneously injected at two sites with 200 μg of rat MOG peptide 35–55 emulsified in complete Freund's adjuvant containing 400 μg of Mycobacterium tuberculosis (Difco Laboratories, Detroit, MI, USA). On the day of (d0) and 2 d after (d2) immunization, the mice were intravenously injected with 200 ng of pertussis toxin (Sigma-Aldrich, St. Louis, MO, USA). All mice were weighed, examined and graded daily for neurological signs in a blinded manner as follows: 0, no disease; 1, decreased tail tone or slightly clumsy gait; 2, tail atony and moderately clumsy gait and/or poor righting ability; 3, limb weakness; 4, limb paralysis; and 5, moribund state. Average disease scores were assessed daily. Additionally, in the EAE model, we documented the weight changes during the disease course. Only mice with a score of at least 2 for more than two consecutive days were judged to have fully developed EAE. The maximum clinical score achieved by each animal during the 30-day-observation period was used to calculate average maximum clinical scores for each experimental group. To study the time course of disease development, average clinical scores were calculated daily for each group of mice and plotted. MOG35–55 induced monophasic EAE was monitored for 30 days. Animals were euthanized if scores were worse than grade 4. Immunological studies were performed on the onset (8–12 day after immunization) or peak (19–21 day after immunization) of disease. Mice were chosen according to the typical and representative clinical symptoms.

#### Adoptive Transfer EAE Model

To prepare encephalitogenic cells for adoptive transfer of EAE, mice were immunized with MOG35-55 as described above. Spleens and lymph nodes were collected and cell suspensions were prepared after 10 days of induction. Cells (6 × 10^6^/ml) were cultured in RPMI 1640 medium (supplemented with 10% fetal bovine serum, 2 mM L-glutamine, 1 mM sodium pyruvate, 100 IUml^−1^ penicillin/streptomycin and 2 × 10^−5^M 2-ME [Life Technologies, Carlsbad, CA, USA)], with MOG35–55 (20 μg/ml), and IL-12 (30 ng/ ml) (R&D Systems, Minneapolis, MN, USA). After 3 days in culture, the cells were harvested, washed in phosphate-buffered saline (PBS) and injected into recipient mice intravenously that were irradiated sublethally (500 rad) within 16 h before cell injection. All mice were weighed, examined, and scored daily after cell transfer (24).

#### Treatment of Bindarit

2-[(1-benzyl-indazol-3-yl) methoxy]-2-methyl propionic acid (bindarit) is a small synthetic indazolic derivative that preferentially inhibits transcription of CCL-2 (25). Bindarit has been shown some clinical efficacy in treating a broad array of experimental inflammatory, autoimmune and vascular disorders; it also had some success in recent clinical trials for diabetic nephropathy and lupus nephritis (26).

The method for bindarit treatment in animals has been previously described (26). Briefly, bindarit was prepared as a suspension in dimethyl sulfoxide (DMSO) at a concentration of 40 mg/ml. Bindarit (200 mg/kg) or vehicle DMSO were given i.p. on the day before EAE induction (day-1) for three consecutive days, and then every other day. This schedule was designed to minimize trauma associated with daily injections at times of peak neurologic disease and physical compromise.

#### Treatment of Anti-IL-6

For IL-6 blockade experiments, mice were i.p. treated with 200 μg of LEAF™ Purified anti-mouse IL-6 (clone MP5-20F3, rat IgG1) or the isotype control antibody Rat IgG1(clone RTK2071) (BioLegend, San Diego, CA, USA) on Day−1 (27).

### Immunohistochemistry and Immunofluorescence Staining

For immunohistochemical (IHC) analysis of spinal cord tissues, mice were euthanized at the peak of EAE by intracardiac perfusion with ice-cold PBS, followed by 4% paraformaldehyde solution, under anesthesia. Spinal cords were rapidly dissected and sectioned at a thickness of 25 μm. For detecting inflammatory infiltrates, the lumbar sections were stained with hematoxylin and eosin (HE).

For immunofluorescence staining, lumbar sections were rinsed in PBS, blocked by incubation with 1% bovine serum albumin at 37°C for 1 h, then incubated overnight at 4°C with primary antibodies (rat anti-mouse CD45 antibody, 1/1,000; Goat anti-mouse IBA1 antibody, 1/1,000; rabbit anti-CCL-2 polyclonal antibody, 1/200; anti-mouse IL-6 antibody, 1/500). The sections were incubated with appropriate FITC secondary antibodies at 37°C for 1 hour. All antibodies were diluted in 1% bovine serum albumin in PBS. The bright field images were taken on a BX51 Olympus microscope (Olympus Corporation, Tokyo, Japan); Immunofluorescent images were recorded using a Zeiss LSM 510 Meta confocal microscope (Carl Zeiss MicroImaging Inc., Thornwood, NY, USA). For the quantification, five sections from each mouse were used for cell counting. Cells were counted using ImageJ (US National Institutes of Health) in a designated area. Data represent mean ± Standard Deviation (SD) of 5 mice for each group.

### Isolation of Leukocytes From Spleen/Lymph Nodes and Brain

Spleens and draining lymph nodes were harvested, minced in PBS, and pushed through a 70-μm mesh. Red blood cells were lysed and cells were collected. Brains were homogenized in ice-cold tissue grinders, filtered through a 70-μm cell strainer and the cells were collected by centrifugation at 400 g for 5 min. Cells were resuspended in 10 ml of 30% Percoll (Amersham Bioscience) and centrifuged onto a 70% Percoll cushion in 15-ml tubes at 800 g for 30 min. Cells at the 30–70% interface were collected and were subjected to flow cytometry.

### T Cell Proliferation

To examine the proliferation of T cells, we isolated spleen and lymph nodes from MOG35–55-immunized mice in the onset and peak of EAE and cultured T cells in a 96-well plate (1 × 10^5^ per well) in the presence of MOG35–55 (0, 0.8, 4, 20 and 100 μg/ml) or Con A (10 μg/ml) (Sigma-Aldrich, St. Louis, MO, USA). Cells were maintained in RPMI 1640 medium supplemented with 10% fetal bovine serum (Life Technologies, Carlsbad, CA, USA), 2 mM L-glutamine, 1 mM sodium pyruvate, 100 IU/ml penicillin/streptomycin and 2 × 10^−5^ M 2-ME (Life Technologies, Carlsbad, CA, USA) for 72 h. Cell proliferation was determined using an AMR PLUS kit (Lonza Rockland, Rockland, ME, USA) according to manufacturer's instruction. The kit can be used for the direct assessment of cell numbers as each individual cell contains ATP. The bioluminescence was analyzed with a luminometer (Bio-Tek, Atlanta, GA, USA).

For serum stimulation experiment, serum was collected from mice which were fed on ND or HFD for 8 weeks. Immune cells were isolated from spleen and lymph nodes of control wild-type mice (CT) and EAE mice which were immunizied with MOG35-55 after 11 days of induction. The immune cells were then cultured with ND serum or HFD serum in the presence of MOG35–55 (20 μg/ml) for 3 days. Cell proliferation was determined using AMR PLUS kit, the Relative Light Units (RLUS) of bioluminescence was analyzed with a luminometer.

### Flow Cytometry

Cells (1 × 10^6^/ml) obtained from brain, spleen and lymph nodes were washed and resuspended in PBS. Cells were stained for surface markers with specific antibodies in fluorescence-activated cell sorting (FACS) buffer at 4°C for 40 min. Cells were washed twice and resuspended in the 200–400 μl of PBS for flow cytometry analysis as previously described (28, 29). For intracellular staining, cells were incubated in a 96-well plate (1 × 10^6^ per well) with MOG35–55 (20 μg/ml) for 16 h, and PMA(50 ng/ml)/Ionomyocin(1 ug/ml) and Brefeldin A (1: 1000 dilution; BioLegend, San Diego, CA,USA) was added in the last 4–5 h, then cells were collected, fixed and permeabilized using Fixation & Permeabilization Buffer (eBioscience, San Diego, CA, USA), followed by the staining using fluorescence labeled rat anti-mouse antibodies directed against IFN-γ or IL-17. Cells were then analyzed with FortessaX20 (BD Biosciences, San Diego, CA, USA). Data were analyzed with FlowJo software (Tree Star, San Carlos, CA, USA).

### Statistical Analysis

Statistical analysis was assessed by ANOVA followed by Student–Newman–Keuls analyses when more than two groups were compared, and *t*-test was used when only two groups were compared. An unpaired *t*-test was used for the analysis of quantitative data of cell counting. Data were presented as means ± SD. Difference in which *p* < 0.05 was considered statistically significant.

## Results

### HFD Exacerbates EAE in Active Immunization Model

**Figure 1:**
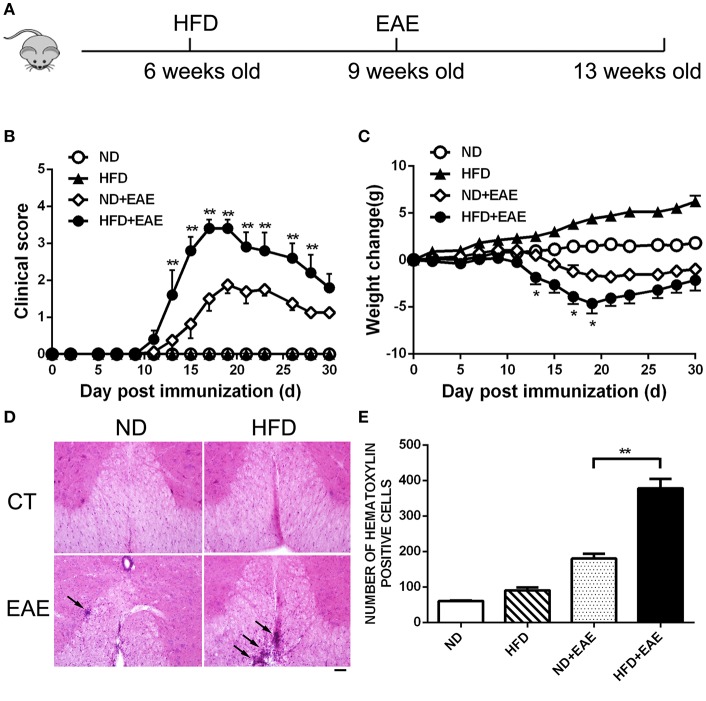
HFD exacerbates EAE in active immunization model. **(A)** C57BL/6 mice were fed on high fat diet (HFD) starting from 6 weeks old, then were immunized with MOG35-55 to induce EAE at 9 weeks old. The clinical score **(B)** and body weight **(C)** was determined in these mice. Data were presented as mean ± SEM; *p < 0.05, **p < 0.01, compared with ND+EAE mice; *n* = 20 for each group. **(D)** The infiltration of inflammatory cells in the spinal cord was detected by hematoxylin and eosin staining on the peak of EAE. Bars = 50 μm. **(E)** The number of hematoxylin positive cells in the spinal cord was quantified using ImageJ software in a designated area. Data were presented as mean ± SD; **p < 0.01, compared with EAE mice; *n* = 5 for each group.

To determine the effect of obesity on the development of EAE, we immunized mice fed on HFD (high-fat diet) for 3 weeks with MOG35–55 peptide to induce an active EAE model. Mice were kept on HFD feeding during the whole course of the disease (Figure 1A). After 11–19 days of immunization, mice developed a monophasic EAE disease characterized by ascending paralysis. Interestingly, the EAE mice fed on HFD showed markedly more severe neurologic dysfunction than control mice fed on ND (Normal Diet). As shown in Table 1, HFD-fed mice had an earlier onset of EAE at day 11.67 ± 1.15 compared with ND-fed mice at day 14.43 ± 2.23, and a higher maximum clinical score at 3.5 ± 0.58 than ND-fed mice at 1.85 ± 0.69. In addition, HFD-fed EAE mice had enhanced disease severity with higher clinical score during disease progression and more severe body weight loss compared with ND-fed EAE mice (Figures 1B,C). We next performed histopathological analysis on spinal cords of EAE mice. Inflammatory cell infiltration in lumbosacral enlargement was examined by hematoxylin and eosin staining. The number of infiltrated cells in HFD-fed EAE mice was increased dramatically than that in ND-fed EAE mice (Figures 1D,E). Collectively, the above data suggest HFD-induced obesity promotes the development and pathogenesis of EAE.

**Table 1:** EAE in ND and HFD mice.

**Diet**	**Incidence**	**Day of onset (mean ± s.d.)**	**Maximum clinical score (mean ± s.d.)**
ND	13 of 20 (65%)	14.43 (±2.23)	1.85 (±0.69)
HFD	14 of 20 (70%)	11.67 (±1.15)	3.5 (±0.58)**

Results are cumulative data from three different experiments,

** *P < 0.01, as compared to corresponding control*.

### HFD Enhances Inflammatory Cells Infiltration in CNS

**Figure 2:**
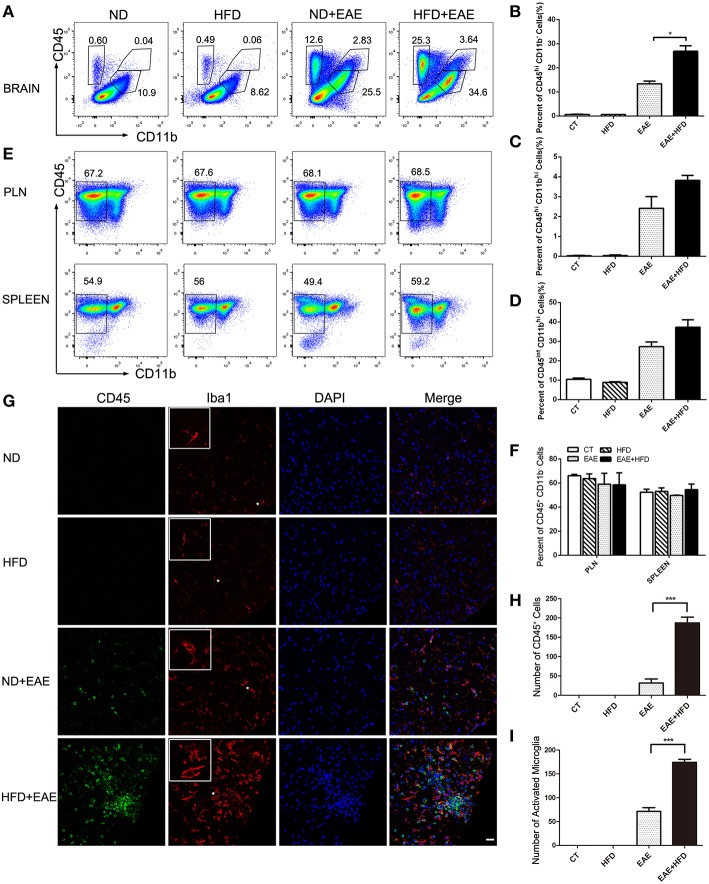
HFD enhances microglial activation in EAE mice. Immune cells infiltration in the brains of MOG35-55-immunized mice fed on ND and HFD was determined at the peak of disease by flow cytometry with antibodies to the indicated cell markers. The percentage of invading cells (CD45^hi^CD11b^−^) cells, macrophages (CD45^hi^CD11b^hi^) and microglia cells (CD45^int^CD11b^hi^) were analyzed **(A–D)**. Immune cells from lymph nodes and spleen were also determined at the peak of disease by flow cytometric analysis **(E,F)**. The activation of microglia was measure by immunofluorescent staining with an antibody directed against CD45 or Iba-1 **(G)** on day 20 after MOG35-55 immunization. Bars = 20 μm. The number of CD45^+^ cells **(H)** and active microglia **(I)** was determined. Data were presented as mean ± SD; *p < 0.05, ***p < 0.001, compared with EAE mice; *n* = 5 for each group.

EAE is a chronic inflammatory disease in central nervous system (CNS). To determine the extent of CNS inflammation, we examined the infiltrated inflammatory cells in CNS by flow cytometry. HFD induced a significant accumulation of CD45^hi^CD11b^−^cells (invading cells) in CNS (*p* = 0.037). CD45^hi^CD11b^hi^ cells (macrophages) (*p* = 0.084) and CD45^int^CD11b^hi^ cells(microglia) (*p* = 0.233) also showed an increase trend in CNS (Figures 2A–D and Supplementary Figure S1). We also analyzed the systemic inflammation level in spleen and PLN (peripheral lymph nodes) in different group mice at the peak of EAE. No significant differences were observed in the proportion of CD45^+^ CD11b^−^ cells from spleen and PLN (Figures 2E,F). These data indicate that HFD induces more severe inflammation in CNS, but not in systemic level.

We further confirmed the inflammatory cells infiltration in CNS by Immunofluorescent analysis of CD45 and Iba1 (a marker for microglia) in spinal cord (Figure 2G). HFD-fed EAE mice had a significant increase of CD45^+^ cells (491% increase) and Iba1^+^ cells (144% increase) infiltration compared with ND-fed EAE mice (Figure 2H), which was consistent with the previous flow cytometry data. In addition to the number increase, the microglia cells had a more active phenotype in HFD-fed EAE mice as indicated by a larger cell body and thicker processes (Figure 2I). Taken together, HFD enhanced the inflammatory cells infiltration in CNS and microglia activation upon EAE induction.

### HFD Induces Pathogenic CD4^+^ T Lymphocytes Infiltration in CNS

**Figure 3:**
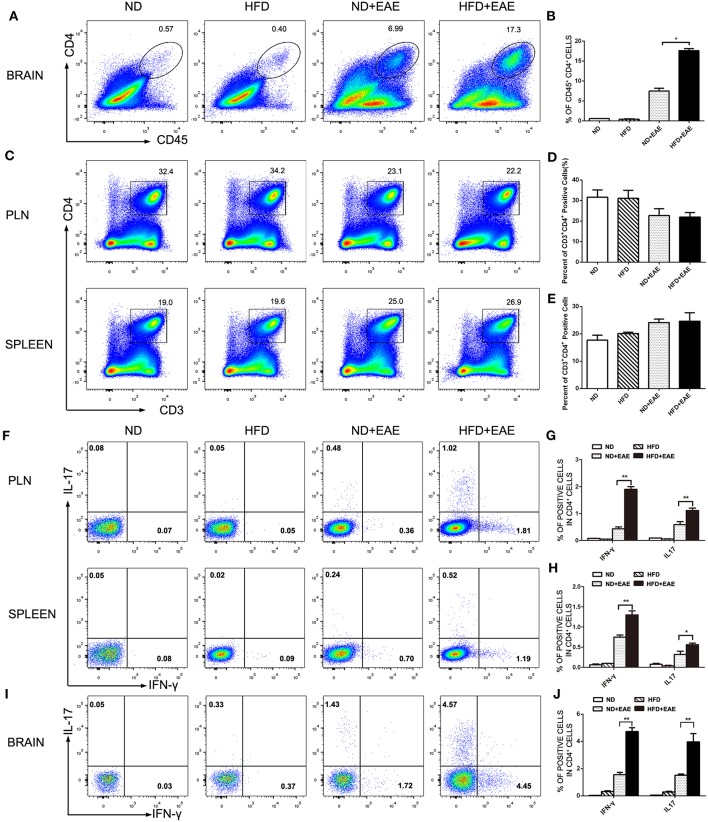
HFD enhances CD4 lymphocytes infiltration in brain. Immune cells were isolated from brain, lymph node and spleen of EAE mice on ND or HFD at the peak of disease. The percentage of CD4^+^ T cells in each tissue was measured by flow cytometry **(A–E)**. To determine Th1 and Th17 responses, cells were stimulatd with MOG35–55 (20 μg/ml) for 16 h, with PMA (50 ng/ml) and Ionomyocin (1 ug/ml) for 5 h in the presence of Brefeldin A and subject to intracellular staining. The percentage of IFN-γ and IL-17-producing cells gated on CD4^+^ T cells were shown **(F–J)**. Data were presented as mean ± SD; *p < 0.05, **p < 0.01, compared with EAE group. *n* = 4 for each group.

The presence of myelin protein-specific autoreactive CD4^+^ Th cells is an immunological hallmark of EAE. The antigen-specific Th1 and Th17 cells are considered to contribute to the pathogenesis of EAE. We further investigated the percentage of CD4^+^ Th cell subsets, as well as functional cytokine production from Th1 and Th17 cells of brain by flow cytometry. Leukocytes were isolated from brain in the peak of EAE. The percentage of total CD4^+^ T cells was increased significantly in EAE mice fed on HFD compared with EAE mice fed on ND (Figures 3A,B). In consistent with the previous findings of total CD45^+^ cells infiltration, there was no obvious difference in the percentage of CD3^+^CD4^+^ T cells infiltration in spleen and PLN between different groups of mice on the peak of EAE (Figures 3C–E). In addition to the cell number, we also determined the activation status of the infiltrated CD4^+^ T cells by measuring the expression level of activation marker CD69, CD25 and CD44 in spleen and LN. There was no significant difference in terms of the activation status of CD4^+^ T cells in spleen and PLN between ND-EAE and HFD-EAE group (Supplementary Figures S2, S3).

IFN-γ-producing Th1 cells and IL-17-producing Th17 cells have been implicated in EAE pathogenesis. Therefore, we analyzed Th1 and Th17 cells induction in the lymph node, spleen and brain using flow cytometry at the peak of EAE. The percentage of Th1 and Th17 cells in EAE mice fed on HFD was enhanced dramatically compared to EAE mice fed on ND in lymph node and spleen (Figures 3F–H), and the increase was even more substantial in the brain (Figures 3I,J). These data suggest that HFD enhanced EAE disease through induction of pathogenic Th1 and Th17 cells.

### HFD Enhances Antigen-Stimulated Proliferation of T Lymphocytes

**Figure 4:**
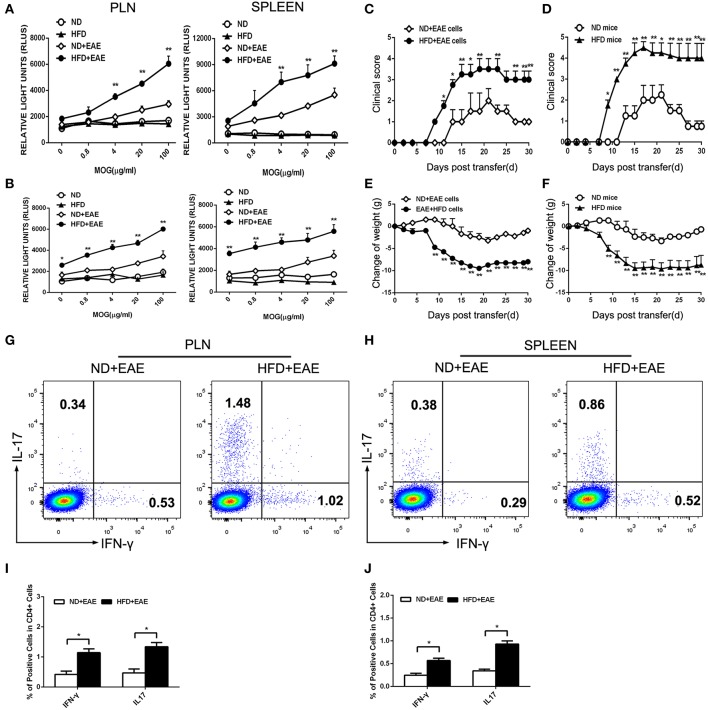
HFD enhances the activation of T lymphocytes in response to MOG35–55. T lymphocytes were isolated from the draining lymph nodes and spleen of mice immunized with MOG35-55 at the onset **(A)** and peak **(B)** of disease followed by *ex vivo* restimulation in the presence of MOG35–55. Cell proliferation was determined using AMR PLUS kit for the assessment of cell numbers, the Relative Light Units (RLUS) of bioluminescence was analyzed with a luminometer. Data were presented as mean ± SD; *p < 0.05, **p < 0.01, compared with ND+EAE group; *n* = 4 for each group. Encephalitogenic cells isolated from EAE mice either on ND or HFD were transferred to the recipient mice WT mice fed on ND. The recipient mice were irradiated sublethally (500 rad) 16 h before cells adoptive transfer. The clinical scores for EAE and body weight were determined **(C,E)**. Encephalitogenic cells from EAE mice were transferred to the recipient mice fed on either ND or HFD. The clinical scores for EAE and body weight were determined **(D,F)**. Lymph node **(G,I)** and spleen **(H,J)** cells were isolated from EAE mice fed on ND or HFD after 11 days of induction. Cells were cultured in RPMI 1640 medium, with MOG35–55 (20 μg/ml) and IL-12 (30 ng/ ml) for 3 days, then the percentage of IFN-γ and IL-17 producing cells were measured by FACS. Data were presented as mean ± SD; *p < 0.05, **p < 0.01, compared with control group. *n* = 8 for each group.

We observed more T lymphocytes infiltrated in brain, which might result from enhanced T cell proliferation or increased T cell migration into CNS. To address this question, we examined the antigen-stimulated proliferation of T lymphocytes isolated from the peripheral lymph node and spleen on the onset and peak of EAE. MOG did not stimulate the proliferation of T lymphocytes isolated from control group ND mice and HFD mice, but significantly increased the proliferation of T lymphocytes isolated from EAE mice. More importantly, the lymphocytes isolated from EAE mice fed on HFD displayed a much stronger and concentration-dependent response to MOG-mediated proliferation which means HFD can obviously potentiates antigen-stimulated proliferation of T lymphocytes in EAE mice (Figures 4A,B).

Despite the higher proliferation activity of T cells from EAE mice fed on HFD, we next questioned whether these antigen-specific T cells would cause more severe EAE. To test this hypothesis, we transferred encephalitogenic cells isolated from EAE mice fed on ND or HFD to recipient mice which were fed on ND and irradiated sublethally. The mice received the encephalitogenic cells isolated from HFD-fed EAE mice developed more severe disease than those received the cells from ND-fed EAE mice, as indicated by higher clinical scores and a greater loss of body weight (Figures 4C,E). As Th1 and Th17 cells are the major pathogenic cells mediating EAE, the proportion of Th1 and Th17 in encephalitogenic cells were analyzed by flow cytometry. There was an obvious increase in the IFN-γ producing cells (*p* = 0.048 for LN; *p* = 0.040 for spleen) and IL-17 producing cells (*p* = 0.044 for LN; *p* = 0.017 for spleen) isolated from EAE mice fed on HFD (Figures 4G,H), which was considered to contribute to the worsened disease. To further determine whether HFD has a direct effect on the encephalitogenic cells, we transferred the encephalitogenic cells isolated from EAE mice to different recipient mice which were fed on ND or HFD, respectively. In HFD-fed recipient mice, the encephalitogenic cells resulted in a quicker and more severe development of EAE than in ND-fed recipients, as determined by the clinical scores. Consistently, the encephalitogenic cells induced greater body weight loss in the HFD-fed recipient mice (Figures 4D,F). To determine whether the enhanced EAE in HFD recipient was mediated by transferred T cells, we isolated lymphocytes from spleen and LN of onset EAE mice and cocultured them with serum from HFD mice and ND mice, respectively, in the presence of MOG35-55. The data showed the EAE T cells had increased proliferation activity upon stimulation with HFD serum compared to ND serum. The results suggested that HFD recipients provided an immune-enhancing factors to promote transferred T cells proliferation, which contributed to the enhanced EAE disease (Supplementary Figure S4). Collectively, the above results suggest that HFD induces more pathogenic T cells in EAE mice than ND.

### CCL-2 and IL-6 Are Involved in HFD-Induced Exacerbation of EAE

**Figure 5:**
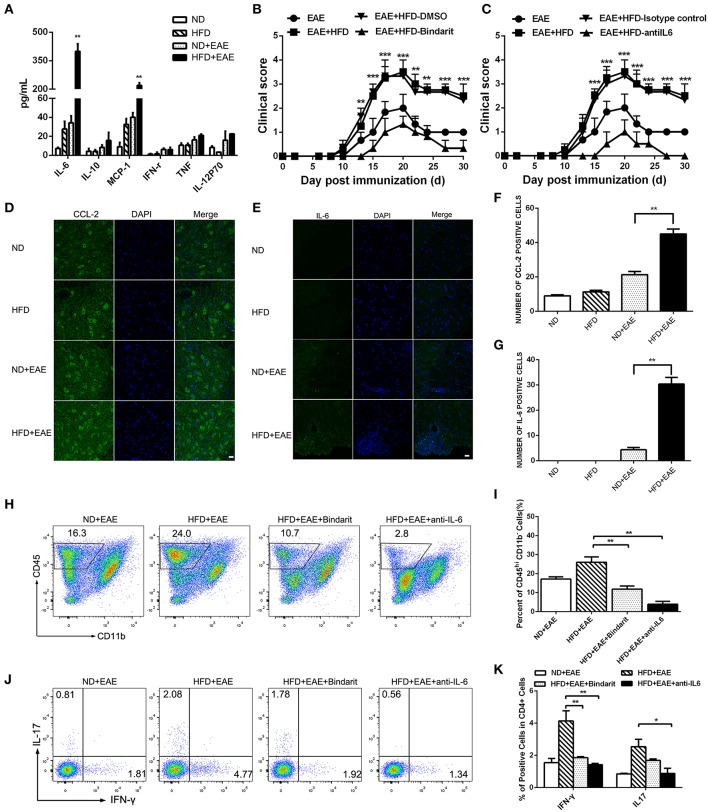
HFD enhances the expression of IL-6 and CCL-2 in EAE mice. The serum was collected from mice in different groups. IL-6, IL-10, CCL-2, IFN-γ, TNF-α and IL-12P70 were detected using BD™ Cytometric Bead Array (CBA) Mouse Inflammation Kit **(A)**. Data were presented as mean ± SEM; **p < 0.01, compared with EAE mice. EAE in C57BL/6 mice was induced by injection of MOG35-55. On day 20 after MOG35-55 injection, CCL-2 **(D,F)**, and IL-6 **(E,G)** were detected in the spinal cord by immunofluorescent staining with an antibody directed against CCL-2 or IL-6. Bars = 20 μm. EAE mice were given daily i.p. injection of bindarit or anti-IL-6 antibody as described under the Materials and Methods. The clinical score **(B,C)** was determined in these mice. Data were presented as mean ± SEM; **p < 0.01, ***p < 0.001 compared with EAE+HFD-DMSO or EAE+HFD-Isotype control mice; *n* = 8 for each group. The percentage of CD45^+^ cells **(H,I)**, IFN-γ+ and IL-17+ Th cells **(J,K)** were measured by FACS. Data were presented as mean ± SD; n = 5 for each group. *p < 0.05, **p < 0.01, compared with HFD+EAE mice.

Obesity-associated chronic inflammation is considered as a potent accelerator for many inflammatory diseases. The inflammatory state in obesity is characterized by many elevated pro-inflammatory cytokines. In order to determine which factors are responsible for the exacerbation of EAE mice fed on HFD, we collected the serum in the peak of EAE and performed cytokine array analysis including IL-6, IL-10, CCL-2, IFN-γ, TNF-α and IL-12P70 using BD™ Cytometric Bead Array (CBA) Mouse Inflammation Kit. Interestingly, the expression level of IL-6 and CCL-2 was markedly increased in HFD-fed mice compared with ND-fed control mice. In order to determine whether the increased IL-6 and CCL2 are the causal factors of enhanced EAE in HFD mice, we collected the serum of mice fed on HFD for 4 weeks before EAE onset and determined the level of proinflammatory cytokines of IL-6 and CCL2 by BD™ Cytometric Bead Array (CBA). As shown in Supplementary Figure S5, both IL-6 and CCL2 were increased significantly compared to ND mice. In the further, the expression of IL-6 and CCL-2 was increased more significantly in HFD-fed EAE mice. However, other cytokines showed the comparable expression level (Figure 5A). The results indicated that IL-6 and CCL-2 might play a very important role in the process of HFD exacerbating EAE. To further confirm their effect on CNS inflammation, we examined the expression level of IL-6 and CCL-2 in the spinal cord by immunofluorescence (Figures 5D–G). The number of IL-6- and CCL-2-producing cells in CNS was raised substantially in EAE mice fed on HFD compared with EAE mice fed on ND. These data indicate that HFD worsened the CNS inflammation featured by elevated IL-6 and CCL-2 level.

To determine whether elevated CCL-2 contributes to the exacerbation of EAE in HFD-fed mice, we treated the mice with bindarit, an inhibitor of CCL-2 synthesis. We found Bindarit had therapeutic effects on EAE mice fed on HFD by lowering the clinical scores (Figure 5B). HFD-fed EAE Mice showed a rapid progression of EAE, reaching a maximum mean clinical score of 3.45 ± 1. Bindarit-treated HFD-fed EAE mice had a slower progression and a maximum mean clinical score of 1.33 ± 0.57. In consistent with the phenotype of the mice, the proportion of CD45^+^ cells, Th1, and Th17 cells in the brain was decreased significantly in HFD-fed EAE mice treated with bindarit (Figures 5H–K). To assess whether IL-6 has a functional role in promoting the disease, we treated the HFD-fed EAE mice with anti-IL-6 blocking antibody. We found that blockade of IL-6 had therapeutic effects on the EAE mice fed on HFD as well, as indicated by lowering the clinical scores (Figure 5C). HFD-fed EAE mice treated with anti-IL-6 had slower progression and lower clinical score. Similar to the treatment of bindarit, infiltrated inflammatory cells including CD45^+^, Th1, and Th17 cells were all reduced markedly in CNS of EAE mice fed on HFD (Figures 5H–K). Taken together, these data suggest that obesity promotes the pathogenesis of EAE through CCL-2 and IL-6 mediated CNS inflammation.

## Discussion

Recent epidemiological studies have linked the rise of MS with the incidence of obesity (5–8). Obesity is associated with chronic low-grade inflammation characterized by elevated proinflammatory cytokines TNF-α, IL-6, IL-1β, and CCL-2 (18, 19). Thus, the proinflammatory state in obesity may have immune-enhancing effect on the development of the inflammatory diseases, such as MS, Inflammatory bowel disease (IBD) and Rheumatoid arthritis (RA). In this study, we have determined the effect of obesity on the development of autoimmune disease MS using a mouse model of HFD-induced obesity and EAE. We have shown that HFD-fed mice developed an exacerbated EAE with severe clinical and pathological symptoms than the control ND-fed mice. The exacerbation of EAE in HFD mice was associated with enhanced MOG-specific T cell proliferation and increased expansion of Th1 and Th17 cells. Moreover, HFD augmented the expression level of IL-6 and CCL-2 both in serum and brain, and blockade of IL-6 and CCL-2 signal ameliorated EAE with reduced autoreactive T cell responses in CNS. Our study suggested that obesity promotes CNS inflammation in EAE through enhanced autoreactive T cell immune responses mediated by IL-6 and CCL-2.

In our study, we have found that HFD-induced obesity is important for the activation and proliferation of MOG35-55-reactive effector T cell population in the secondary lymphoid organs. Th1 and Th17 cells are the two major effector cell subsets required for the pathogenesis of EAE. Their activation results in the production of the effector cytokines IFN-γ and IL-17, which are capable of inducing macrophage activation and neutrophil recruitment to the inflammatory sites to accelerate the lesions. Given the critical role of Th1 and Th17 CD4^+^ cells in the progression of EAE, the increase of both Th1 and Th17 are considered to contribute the worsen disease in HFD-fed EAE mice of our study. In the adoptive transfer of EAE model, encephalitogenic cells isolated from HFD-fed mice were more pathogenic than those from ND-mice. We found both IFN-γ-producing Th1 and IL-17-producing Th17 cells were significantly increased in the encephalitogenic cells from HFD-fed mice, indicating Th1 and Th17 cells account for the disease exacerbation. HFD-fed EAE mice exhibited not only the expansion of the pathogenic T cells, but also the enhanced proliferation of the MOG-specific T cell responses. The above data indicate that obesity has an effect of enhancing antigen-specific T cell immune responses.

In this study, we also observed that HFD could enhance microglial activation in CNS. Like macrophages, activated microglia cells have two polarization states, M1, and M2. Upon IFN-γ stimulation, microglia cells polarize toward M1 state and release of IL-1, IL-6, IL-12, IL-23, and inducible nitric oxide synthase (iNOS). In addition, M1 microglia can produce diverse chemoattractant chemokines, such as CCL-2 and CXCL10 (30, 31). Therefore, M1 microglia cells play critical roles in promoting CNS inflammation through production of the pro-inflammatory factors. In contrast, IL-4, and IL-13 can turn microglia into M2 cells, which promote tissue repair through production of IL-10 and arginase 1 (32, 33). In our study, we have found HFD mice had increased level of IL-6 and CCL-2, while no change in IL-10 level in the serum. Both IL-6 and CCL-2 are mainly produced by M1 microglia, which indicate microglia cells are more like M1 activation state in our HFD mice. Meanwhile, M1 microglia cells take part in the attraction and differentiation of pathogenic Th1/Th17 (34). Th1/Th17 activation results in the production of the effector cytokines IFN-γ and IL-17 which further polarize microglia toward the M1 state. This forms a positive feedback loop.

Previous studies support the notion that obesity-associated inflammation is the major driver for the insulin resistance and Type 2 diabetes (18–20). The systemic chronic inflammation is featured by increased TNF-α, IL-1-β, IL-6, and CCL-2. These obesity-related proinflammatory cytokines might contribute to the aggravation of EAE in HFD-fed mice. Among those cytokines, we found that IL-6 and CCL-2 were markedly increased in the serum and brain of HFD EAE mice compared to the ND EAE mice, indicating the potential role of IL-6 and CCL-2 in promoting EAE. Further blockade of IL-6 and CCL-2 signaling pathway leads to the amelioration of EAE in HFD mice with decreased pathogenic Th cells infiltration in CNS. Taken together, these results suggest that IL-6 and CCL-2 are the inflammatory mediators linking obesity with enhanced effector Th responses and EAE.

IL-6 is an important mediator in various immunological and inflammatory processes that play a role in the pathogenesis of CNS disorders. It is a regulator of Th17 differentiation *in vitro* and a potential target for the inhibition of Th17 development *in vivo*. IL-6-deficient mice have been shown to be highly resistant to the induction of EAE (35, 36). Previous studies have revealed that IL-6 dependent Th17 expansion exacerbates EAE (37). In contrast, chronic calorie restriction in obese mice can attenuate EAE severity through decreased level of IL-6 production (38). In consistent with these findings, we found that HFD EAE mice exhibited increased IL-6 level and enhanced Th17 responses in both peripheral and CNS. Anti-IL-6 monoclonal antibody treatment resulted in decreased IL-17A^+^ CD4 T cell population in HFD EAE mice CNS. Particularly, IFN-γ-producing Th1 cells in HFD EAE mice CNS were reduced as well. This finding aligns with the report that demonstrates the plasticity of Th17 cells during EAE pathogenesis (39). They show that classical Th1 cells were present at 1:1 ratio with Th17 cells in the draining lymph nodes of EAE. After entering in CNS, classical Th1 cells rapidly disappeared. But some Th17 cells were converted to IL-17 IFNγ^+^cells, so-called ex-Th17 cells. These IFNg-producing ex-Th17 cells became the major Th1 subsets in CNS, and could be inhibited by anti-IL-6 as well. Therefore, we found that both Th1 and Th17 cells were reduced after IL-6 blockade.

In addition to IL-6, we found CCL-2 was increased drastically in obesity EAE mice. CCL-2 is secreted mainly by adipocytes and macrophages during obesity. CCL-2 can regulate the activity of monocytes, dendritic cells, and NK cells and plays an important role in innate immunity (40). It is also associated with pathological inflammation (41). CCR2 is a C-C chemokine receptor that responds predominantly to CCL-2. In murine EAE, the expression of CCL-2 mRNA in the brain and spinal cord was upregulated and may mediate the onset of EAE (42, 43). We have previously demonstrated that neuronal CCL-2 plays an important role in microglia recruitment/activation in the brain (44). On the other hand, CCL-2 can also enhance T cell proliferation and migration in EAE (45). In our study, we found both effector Th1 and Th17 cells were reduced in CNS after Bindarit treatment, which is an inhibitor of CCL-2 synthesis. This might be explained by that the migration and proliferation of Th1 and Th17 cells are suppressed by CCL-2 signaling blockade.

Both IL-6 and CCL-2 are proinflammatory cytokines and contribute to the EAE pathogenesis. We observed enhanced IL-6 and CCL-2 production in obesity animals, which derived from adipose tissue M1 macrophages and adipocytes. We have shown that blockade of either of them attenuated EAE pathogenesis of HFD mice. The mechanism of action was different. IL-6 was considered as a key regulator to promote Th17 development and suppress Treg development. Blocking IL-6 in EAE resulted in reduced Th17 responses and CNS inflammation. CCL-2 was involved in the inflammatory monocytes recruitment to CNS and T cells migration to CNS. Blockade of CCL-2 resulted in reduced T cells and macrophages infiltration in CNS. Both of them are critical for EAE pathogenesis. We speculate that combinational therapy will have a synergized effect on EAE. In terms of the relationship between IL-6 and CCL-2, it is very likely that inflammatory monocytes recruited to CNS by CCL-2 could differentiate into M1 macrophages which can produce IL-6 and more CCL-2 to form a positive feedback loop to attract more macrophage into CNS.

Therefore, obesity can cause chronic inflammation with enhanced proinflammatory cytokines IL-6 and CCL-2 production, which have the immune-enhancing effect to boost the effector Th1 and Th17 cell responses in HFD EAE. Other than immune-mediated mechanisms, HFD could also promote EAE severity through activation of the renin- angiotensin system (46).

In summary, our results demonstrate that HFD-induced obesity enhances significantly the severity of EAE. Mechanistically, obesity induces a pro-inflammatory microenvironment featured by elevated CCL-2 and IL-6 levels, and promotes the proliferation and activation of encephalitogenic T cells into the CNS. Our findings lead us to speculate that targeting IL-6 and CCL-2 is a possible therapeutic strategy for inhibition of obesity-associated CNS inflammation.

## Data Availability

All datasets generated for this study are included in the manuscript and/or the Supplementary Files.

## Ethics Statement

All animal experiments were approved by the animal committee of the School of Basic Medical Sciences, Shanghai Jiao Tong University.

## Author Contributions

LS and ZJ designed research, analyzed data, and wrote the main manuscript text. ZJ, SW, and YX performed research. ZJ prepared figures. All authors reviewed the manuscript.

### Conflict of Interest Statement

The authors declare that the research was conducted in the absence of any commercial or financial relationships that could be construed as a potential conflict of interest.

## Supplementary Material

Supplementary Figure S1 Gating strategy of immune cells in the brain. Leukocytes were isolated from brain in the peak of EAE. The percentage of invading cells (CD45^hi^CD11b^−^) cells, macrophages (CD45^hi^CD11b^hi^) and microglia cells (CD45^int^CD11b^hi^) were gated as indicated and determined by flow cytometry.

Supplementary Figure S2 CD4^+^ T cells activation status in HFD EAE mice compared to ND EAE mice. Immune cells were isolated from draining lymph nodes and spleen of EAE mice fed on ND or HFD after 19 days of induction. The percentage of CD25^+^or CD69^+^ cells in total CD3^+^CD4^+^ cells **(a,b)** were measured by flow cytometry. Data were presented as mean ± SD; *n* = 3 for each group.

Supplementary Figure S3 CD44^+^CD4^+^ T cells in HFD EAE mice compared to ND EAE mice. Lymph node and spleen cells were isolated from EAE mice on ND or HFD after 19 days of induction. The percentage of CD44^+^ cells in CD3^+^CD4^+^ cells was measured by flow cytometry. Data were presented as mean ± SD; *n* = 3 for each group.

Supplementary Figure S4 HFD serum enhances the proliferation of T lymphocytes in response to MOG35-55. Serum was collected from mice which were fed on ND or HFD for 8weeks. Immune cells were isolated from draining lymph nodes and spleen of control wild-type mice (CT) and EAE mice which were immunizied with MOG35-55 after 11 days of induction. The immune cells were then cultured with ND serum or HFD serum in the presence of MOG35-55 (20 μg/ml) for 3 days. Cell proliferation was determined using AMR PLUS kit, the Relative Light Units (RLUS) of bioluminescence was analyzed with a luminometer. Data were presented as mean ± SD; ^*^*p* < 0.05, ^***^*p* < 0.001, compared with EAE group; *n* = 3 for each group.

Supplementary Figure S5 HFD increases the level of IL-6 and CCL2 in the serum. The serum was collected from mice fed on ND and HFD for 4 weeks. The level of IL-6 and CCL2 was measured using BD™ Cytometric Bead Array (CBA) Mouse Inflammation Kit. HFD mice had increased level of IL-6 abd CCL2 compared to ND group mice. (*n* = 5, ^*^*p* < 0.05).
