# Supplementary figures and images for: Obesity Promotes EAE Through IL-6 and CCL-2-Mediated T Cells Infiltration
Source: Front Immunol. 2019 Aug 27;10:1881. doi: 10.3389/fimmu.2019.01881 (PMC6718738; doi:10.3389/fimmu.2019.01881)

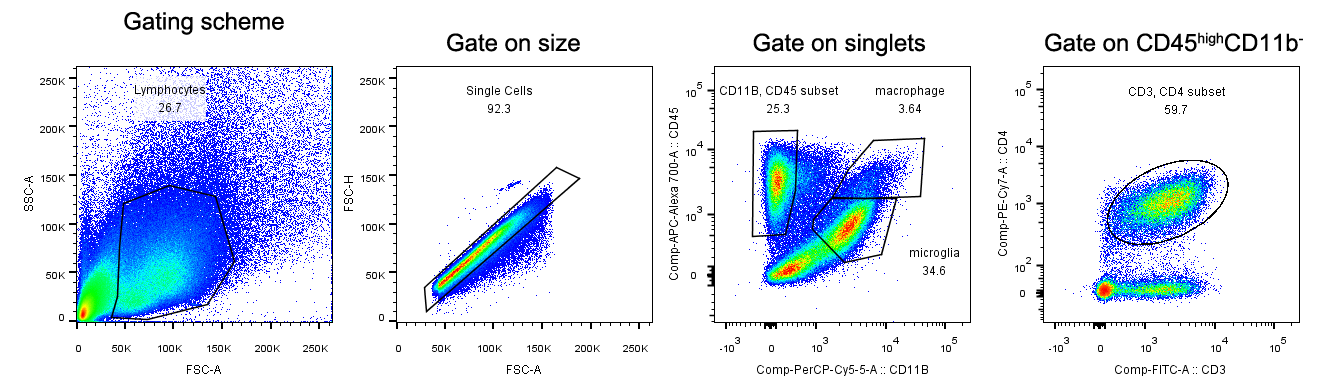

Supplement: Supplementary Figure S1 — Gating strategy of immune cells in the brain. Leukocytes were isolated from brain in the peak of EAE. The percentage of invading cells (CD45hiCD11b−) cells, macrophages (CD45hiCD11bhi) and microglia cells (CD45intCD11bhi) were gated as indicated and determined by flow cytometry. [file Image_1.TIF]

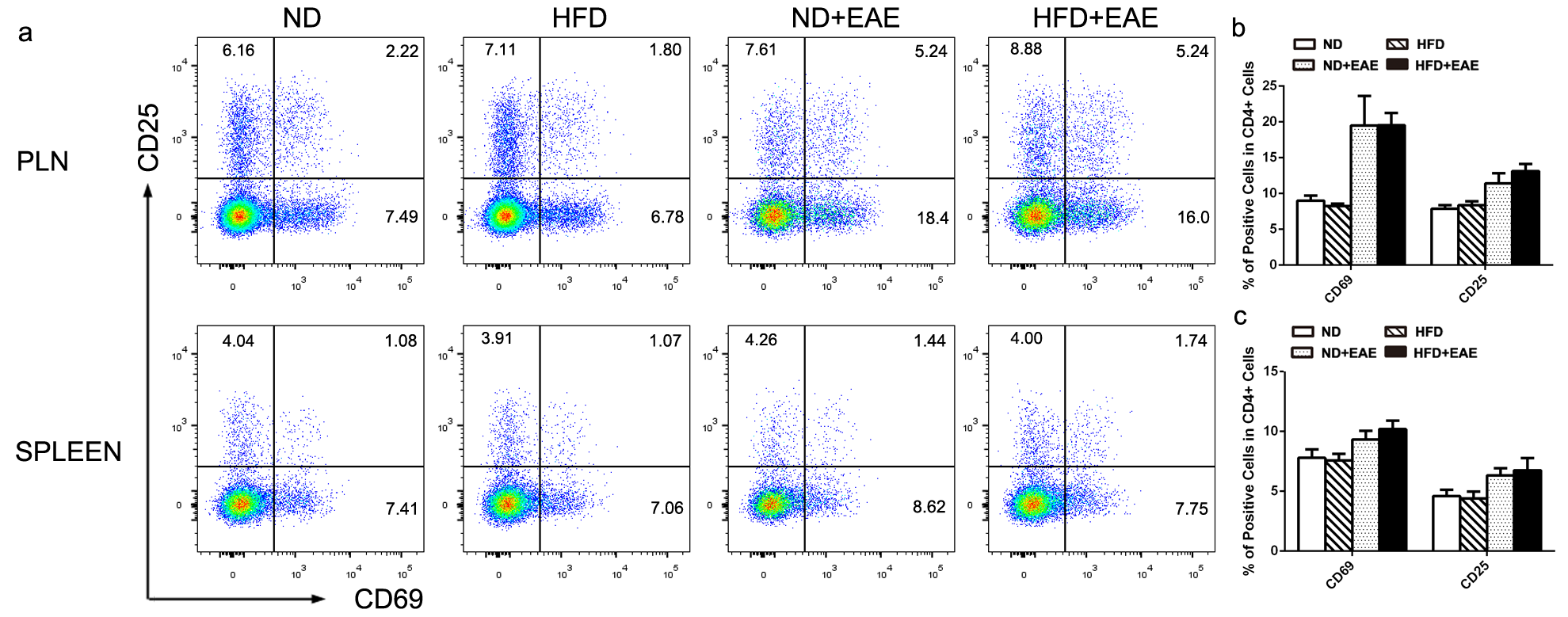

Supplement: Supplementary Figure S2 — CD4+ T cells activation status in HFD EAE mice compared to ND EAE mice. Immune cells were isolated from draining lymph nodes and spleen of EAE mice fed on ND or HFD after 19 days of induction. The percentage of CD25+or CD69+ cells in total CD3+CD4+ cells (a,b) were measured by flow cytometry. Data were presented as mean ± SD; n = 3 for each group. [file Image_2.TIF]

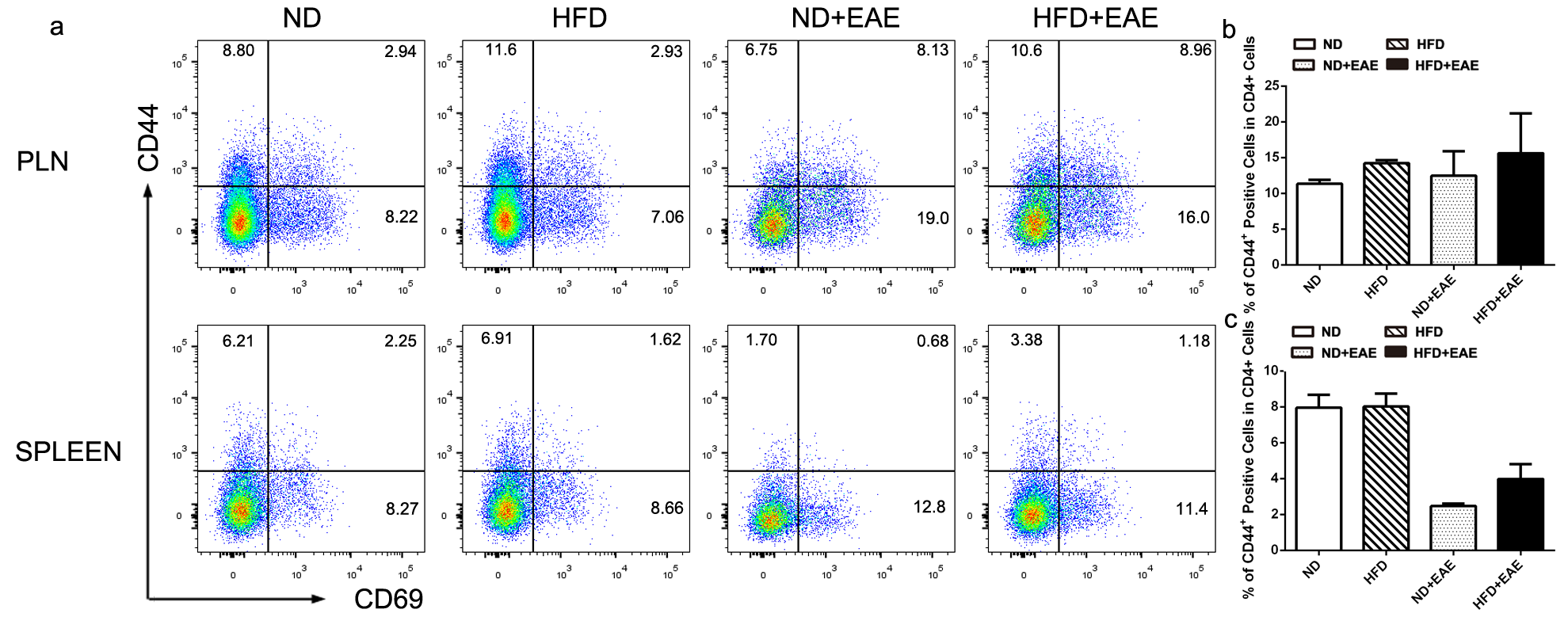

Supplement: Supplementary Figure S3 — CD44+CD4+ T cells in HFD EAE mice compared to ND EAE mice. Lymph node and spleen cells were isolated from EAE mice on ND or HFD after 19 days of induction. The percentage of CD44+ cells in CD3+CD4+ cells was measured by flow cytometry. Data were presented as mean ± SD; n = 3 for each group. [file Image_3.TIF]

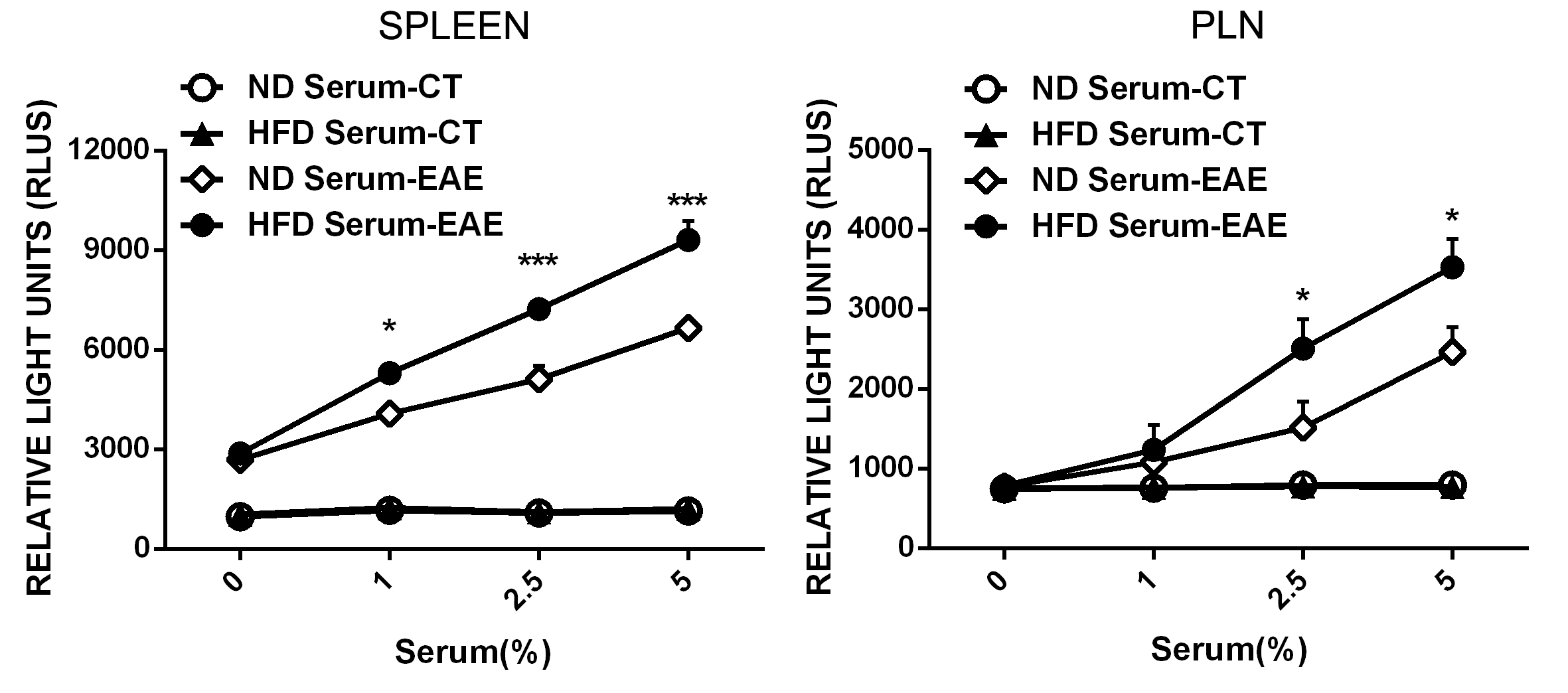

Supplement: Supplementary Figure S4 — HFD serum enhances the proliferation of T lymphocytes in response to MOG35-55. Serum was collected from mice which were fed on ND or HFD for 8weeks. Immune cells were isolated from draining lymph nodes and spleen of control wild-type mice (CT) and EAE mice which were immunizied with MOG35-55 after 11 days of induction. The immune cells were then cultured with ND serum or HFD serum in the presence of MOG35-55 (20 μg/ml) for 3 days. Cell proliferation was determined using AMR PLUS kit, the Relative Light Units (RLUS) of bioluminescence was analyzed with a luminometer. Data were presented as mean ± SD; *p < 0.05, ***p < 0.001, compared with EAE group; n = 3 for each group. [file Image_4.TIF]

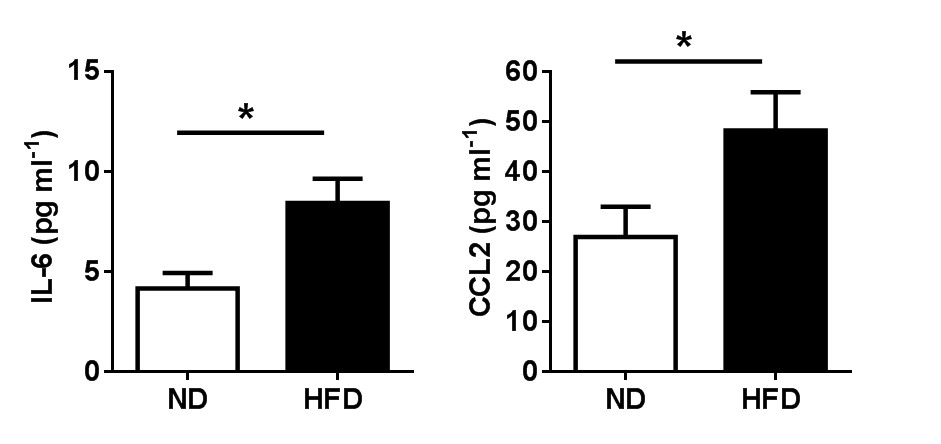

Supplement: Supplementary Figure S5 — HFD increases the level of IL-6 and CCL2 in the serum. The serum was collected from mice fed on ND and HFD for 4 weeks. The level of IL-6 and CCL2 was measured using BD™ Cytometric Bead Array (CBA) Mouse Inflammation Kit. HFD mice had increased level of IL-6 abd CCL2 compared to ND group mice. (n = 5, *p < 0.05). [file Image_5.TIF]
